# Human Wharton's jelly-derived mesenchymal stem cells alleviate concanavalin A-induced fulminant hepatitis by repressing NF-κB signaling and glycolysis

**DOI:** 10.1186/s13287-021-02560-x

**Published:** 2021-09-09

**Authors:** Lijie Pan, Chang Liu, Qiuli Liu, Yanli Li, Cong Du, Xinmei Kang, Shuai Dong, Zhuowei Zhou, Huaxin Chen, Xiaoqi Liang, Jiajie Chu, Yan Xu, Qi Zhang

**Affiliations:** 1 Biotherapy Center, The Third Affiliated Hospital, Sun Yat-Sen University, Guangzhou, 510630 China; 2 Cell-Gene Therapy Translational Medicine Research Centre, The Third Affiliated Hospital, Sun Yat-Sen University, Guangzhou, China; 3 Guangdong Provincial Key Laboratory of Liver Disease Research, The Third Affiliated Hospital, Sun Yat-Sen University, Guangzhou, China

**Keywords:** Fulminant hepatitis, Human umbilical cord Wharton's jelly-derived mesenchymal stem cells (hWJ-MSCs), Immunomodulation, Concanavalin A (Con A), NF-κB, Glycolysis

## Abstract

**Background:**

Fulminant hepatitis is a severe life-threatening clinical condition with rapid progressive loss of liver function. It is characterized by massive activation and infiltration of immune cells into the liver and disturbance of inflammatory cytokine production. Mesenchymal stem cells (MSCs) showed potent immunomodulatory properties. Transplantation of MSCs is suggested as a promising therapeutic approach for a host of inflammatory conditions.

**Methods:**

In the current study, a well-established concanavalin A (Con A)-induced fulminant hepatitis mouse model was used to investigate the effects of transplanting human umbilical cord Wharton's jelly-derived MSCs (hWJ-MSCs) on fulminant hepatitis.

**Results:**

We showed that hWJ-MSCs effectively alleviate fulminant hepatitis in mouse models, primarily through inhibiting T cell immunity. RNA sequencing of liver tissues and human T cells co-cultured with hWJ-MSCs showed that NF-κB signaling and glycolysis are two main pathways mediating the protective role of hWJ-MSCs on both Con A-induced hepatitis in vivo and T cell activation in vitro.

**Conclusion:**

In summary, our data confirmed the potent therapeutic role of MSCs-derived from Wharton's jelly of human umbilical cord on Con A-induced fulminant hepatitis, and uncovered new mechanisms that glycolysis metabolic shift mediates suppression of T cell immunity by hWJ-MSCs.

**Supplementary Information:**

The online version contains supplementary material available at 10.1186/s13287-021-02560-x.

## Background

Fulminant hepatitis is an emergency clinical condition usually caused by hepatitis viral infection or toxin (or drug) exposure that mediates rapid destruction of liver functions. It is an inflammatory lesion of the liver characterized by massive liver parenchyma necrosis followed by excessive immune responses [1]. No specific therapies are currently available for fulminant hepatitis, and orthotopic liver transplantation is the sole established medical approach with long-term effects for end-stage fulminant hepatitis patients. However, practical application of liver transplantation is limited by the desperate shortage of donor organs and high costs.

Mesenchymal stem cells (MSCs) are stem cells found in almost all tissues and organs [2–5]. These cells are frequently recruited at the sites of damage or inflammation and exhibit potential to differentiate into other cell types [6–8]. Moreover, MSCs exhibited potent immunomodulatory functions both in vitro and in vivo and thus demonstrated the therapeutic potential for many inflammatory disorders, such as acute graft-versus-host disease (aGVHD) [9], rheumatoid arthritis [10], inflammatory bowel diseases [11], and so on. MSCs may modulate the immune responses through either direct cell-to-cell contact or paracrine secretion of soluble factors [12–16]. The therapeutic potential of MSCs in the treatment of liver failure has been confirmed in both preclinical and clinical trials [17–19].

Concanavalin A (Con A)-induced fulminant liver injury is a well-documented murine model closely resembling the pathological process of human viral hepatitis and autoimmune liver disease [20–23]. Previous studies showed that bone marrow-derived MSCs [19, 24–30], tonsil-derived MSCs [31], and adipose tissue-derived MSCs [32–35] effectively protect mice from Con A-induced liver injury. However, the source of MSCs in all these tissues is quite limited, and isolating MSCs from these tissues is invasive. MSCs isolated from the Wharton's jelly of human umbilical cord (which is considered as waste material) (hWJ-MSCs) provide an attractive MSCs source alternatively. hWJ-MSCs share a similar morphology, surface markers, tri-lineage differentiation potential, and potent immunomodulatory properties with MSCs from other origins. Moreover, there are sufficient sources (based on the common cell dose frequently administered to the afore-mentioned disorders (1*10^6^ cells/kg of body weight), the amount of hWJ-MSCs isolated from a single donor are frequently sufficient to treat 30 patients), suitable materials, easy isolation and culture procedures, and no ethical or moral restrictions for utilizing hWJ-MSCs [36]. These advantages make hWJ-MSCs an attractive option for stem cell-based therapy.

In this study, the therapeutic effect of hWJ-MSCs on Con A-induced fulminant liver injury was investigated, demonstrating that hWJ-MSCs could efficiently alleviate Con A-induced liver injury. Mechanistically, hWJ-MSCs suppressed the hepatic T cell infiltration and activation by inhibiting NF-κB signaling and glycolysis. The results confirmed that hWJ-MSCs transplantation is a potential therapeutic approach for treating fulminant hepatitis and uncovered new mechanisms for the immunomodulatory functions of hWJ-MSCs.

## Materials and methods

### Animals

C57BL/6J mice were purchased from GemPharmatech Co., Ltd (Nanjing, China) and housed under specific pathogen-free conditions. Experiments were performed with male animals at 6–8 weeks of age under ethical conditions approved by the Institutional Animal Care and Use Committee of The Third Affiliated Hospital of Sun Yat-sen University.

For Con A-induced fulminant hepatitis model, a single dose of Con A (Sigma Aldrich, St. Louis, MO) was administrated at 15 or 20 mg/kg (only for survival analysis) through the tail vein. PBS or 1 × 10^6^ hWJ-MSCs suspended in 200 μL PBS were transplanted intravenously (i.v) 30 min after Con A administration. Liver tissues were collected at indicated time point, and serum was collected at various time points for further analysis.

For live imaging, cells were labeled with 10 µg/ml 1,1-dioctadecyl-3,3,3,3-tetramethylindotricarbocyanine iodide (DiR; AAT Bioquest, USA) for 15 min at 37 °C according to the manufacturer’s instructions. The DiR-labeled MSCs were washed twice with PBS and transplanted into mouse i.v 30 min after Con A administration. Live imaging was conducted at different time points under anesthesia using Xenogen IVIS Lumina II.

### Isolation and culture of human WJ-MSCs

Human umbilical cord collection and processing were conducted after receiving informed consent from all participants included in the study. The hWJ-MSCs were obtained and expanded as previously described with minor modifications [37]. In summary, to isolate hWJ-MSCs, the umbilical cord was dissected into short sections of about 3 cm in length, and the vessels were manually stripped. The Wharton’s jelly was then separated from the umbilical cord and minced into small fragments with an approximate diameter of 2 mm. The minced tissues were digested by collagenase for 1 h and cultured in low-glucose DMEM supplemented with 10% heat-inactivated fetal bovine serum (FBS), 2 mM L-glutamine, and 1% penicillin–streptomycin at 37 °C in a 5% CO_2_ humidified incubator. The medium was changed every three days, and non-adherent cells were discarded by completely changing the medium. Fibroblast-like cells that were considered to be hWJ-MSCs grew around the explants in about ten days. Adherent hWJ-MSCs were detached using 0.125% trypsin–EDTA and passaged at a split ratio of 1:3 when they reached 80–90% confluence. All of the hWJ-MSCs used in this study were characterized by flow cytometry and tri-lineage differentiation. This study was approved by the Institutional Human Ethics Committee of The Third Affiliated Hospital of Sun Yat-sen University.

### Tri-lineage differentiation of hWJ-MSCs

For differentiation to osteoblasts, hWJ-MSCs were seeded into 6-well plates at a density of 4 × 10^3^/cm^2^. After the cells reached 60–70% confluence, the medium was replaced with osteoblast induction medium containing L-DMEM, 10% (v/v) FBS, 2 mM glutamine, 1% penicillin–streptomycin, dexamethasone (0.1 μM, Merck), vitamin C (50 μg/mL, Sigma-Aldrich), and β-glycerol phosphate (10 mM, Sigma-Aldrich). The medium was replaced every three days, monitoring to note cell growth and morphological changes. After three weeks of induction, osteogenic differentiation was confirmed by staining with 0.5% Alizarin Red S (v/v) (Sigma-Aldrich). For differentiation to adipocytes, hWJ-MSCs were seeded into 6-well plates at a density of 4 × 10^3^/cm^2^. After the cells reached 80–90% confluence, the medium was replaced with an adipogenic induction medium containing H-DMEM, 10% (v/v) FBS, 2 mM glutamine, 1% penicillin–streptomycin, dexamethasone (1 μM, Merck), insulin (10 μg/mL, Prospect), isobutylmethylxanthine (IBMX, 0.5 mM, Sigma-Aldrich), and indomethacin (0.2 mM, Sigma-Aldrich). The medium was replaced every three days, monitoring to note cell growth and morphological changes. After the typical lipid droplets appeared, adipogenic differentiation was confirmed by staining with Oil Red O (O0625, Sigma-Aldrich). For chondrocyte differentiation, a pellet culture system was used. Approximately 2.5 × 10^5^ MSCs were placed in a 15-ml polypropylene tube and centrifuged to form a pellet. The pellet was cultured at 37 °C in a 5% CO_2_ humidified incubator in 500 μl of chondrogenic media containing high-glucose DMEM supplemented with 10 ng/ml transforming growth factor-β3 (TGF-β3; Peprotech), 10^−7^ M dexamethasone (Sigma), 50 μg/ml ascorbate-2-phosphate (Sigma), 40 μg/ml proline, 100 μg/ml pyruvate, and 50 mg/ml ITS + Premix (BD Biosciences; final concentrations: 6.25 μg/ml insulin, 6.25 μg/ml transferrin, 6.25 μg/ml sodium selenite, 1.25 μg/ml bovine serum albumin, and 5.35 mg/ml linoleic acid). The medium was replaced every 3–4 days. Twenty-one days later, the pellets were embedded in paraffin, cut into 3-μm sections, and stained with toluidine blue.

### Flow cytometry and cell sorting

Cells were washed and stained with labeled antibody (or isotype control) according to the manufacturer’s instructions. Flow cytometry analysis was performed on either LSR II (BD) or CytoFLEX flow cytometer (Beckman Coulter, Fullerton, CA, USA), and the data were analyzed with FlowJo7.6 software (Treestar, Ashland, OR, USA). Dead cells were excluded by forward light scatter or forward light scatter plus Zombie Aqua™^.^ Fixable Viability Kit (biolegend, 423102). For fluorescent-activated cell sorting, cells were stained with the appropriate antibodies and isolated on a BD influx cell sorter (BD Biosciences, USA). Antibodies are listed in Additional file 2: Table S1.

### Co-culture of hWJ-MSCs with T cells and the proliferation assay

Human peripheral blood mononuclear cells (PBMC) were isolated by density gradient centrifugation. The T lymphocytes were then stained with CD3-FITC and sorted on a BD influx cell sorter (BD Biosciences, USA). For co-culture, hWJ-MSCs were seeded in 24-well plates and co-cultured with T cells at the ratio of hWJ-MSCs: CD3^+^ T cells = 1:5. The stimulant was phytohaemagglutinin (PHA, Sigma-Aldrich) at a final concentration of 2.5 μg/mL. Following co-culture for 48 h, CD3^+^ T cells stimulated with PHA and the CD3^+^ cells co-cultured with hWJ-MSCs in the presence of PHA were collected and analyzed using CytoFLEX flow cytometer (Beckman Coulter).

### Hematoxylin and eosin (H&E) and Picrosirius red (PSR) staining

Mouse liver tissues at the equivalent locations were fixed in 4% paraformaldehyde overnight and embedded in paraffin. Sections of 4 µm were prepared for H&E or PSR (ab150681, abcam) staining.

### Serum Alanine Aminotransferase (ALT) and Aspartate Transaminase (AST) measurements

Mouse blood was sampled at various time points after Con A administration and then centrifuged at 3000 rpm at 4 °C for 15 min to separate the serum. ALT and AST levels were measured using Hitachi 7020 automatic biochemical analyzer (Hitachi, Tokyo, Japan).

### Isolation of hepatic lymphocytes

Mouse liver tissues were collected and perfused with collagenase I to dissociate cells, followed by filtration through a 200-µm cell strainer. Hepatocytes were removed by centrifuging at 50 g for 3 min at 4 °C. Supernatants were collected and liver non-parenchymal cells were isolated with Percoll (GE Healthcare Bio-Sciences AB, Uppsala, Sweden). Cells were then resuspended in 40% Percoll, underlaid with 60% Percoll, and centrifuged for 20 min at 2,000 rpm with no brakes. Cells at the interface were collected, washed, and counted for further analysis.

### RNA sequencing

The sample preparation, sequencing, and data analysis were performed as previously described [38]. GSEA was conducted using the Gene set enrichment analysis (GSEA) software (https://www.broadinstitute.org/gsea/) [39]. Differentially expressed genes (DEGs) were identified using DESeq2 [40] on the following cutoff criteria: *p* < 0.05 and |Fc|> 1.5**.** GO and KEGG pathway analyses of the DEGs were performed using the online Database for Annotation Visualization and Integrated Discovery (DAVID) (https://david.ncifcrf.gov/), with *p* < 0.05 as the threshold for statistical significance. Sequencing data are available in the Gene Expression Omnibus (GEO) database under the accession number GSE166327 and GSE182182.

### Western blot

Approximately 20 mg of liver tissues was homogenized and lysed with 500 μl lysis buffer containing 62.5 mM Tris–HCl (PH 6.8), 2% SDS, 10% glycerol, 0.02% bromophenol blue, 50 mM DTT, and proteinase inhibitors. Proteins were separated by sodium-dodecyl-sulfate–polyacrylamide gel electrophoresis and transferred to nitrocellulose membrane, blocked with TBS/T containing 5% non-fat dry milk, and analyzed for target proteins with specific primary antibodies listed in Additional file 2: Table S1.

### Statistical analysis

All results were expressed as mean ± SD. Statistical comparison was made using the two-tailed Student’s *t*-test between two groups or one-way ANOVA for multi-group comparison. *p* < 0.05 was considered significant. Analyses and graphs were performed using GraphPad Prism version 7 (San Diego, CA).

## Results

### hWJ-MSCs effectively protected mice from Con A-induced fulminant hepatitis

Like MSCs of other origins and previous reports [2, 3], hWJ-MSCs were strongly positive for CD29, CD73, CD105, and CD90, whereas negative for hematopoietic stem cell markers such CD34 and CD45 (Additional file 1: Figure S1A). The cells showed typical spindle-shaped fibroblastic morphology (Additional file 1: Figure S1B) and were successfully induced to differentiate into osteoblasts, adipocytes, and chondrocytes (Additional file 1: Figure S1C).

**Fig. 1:**
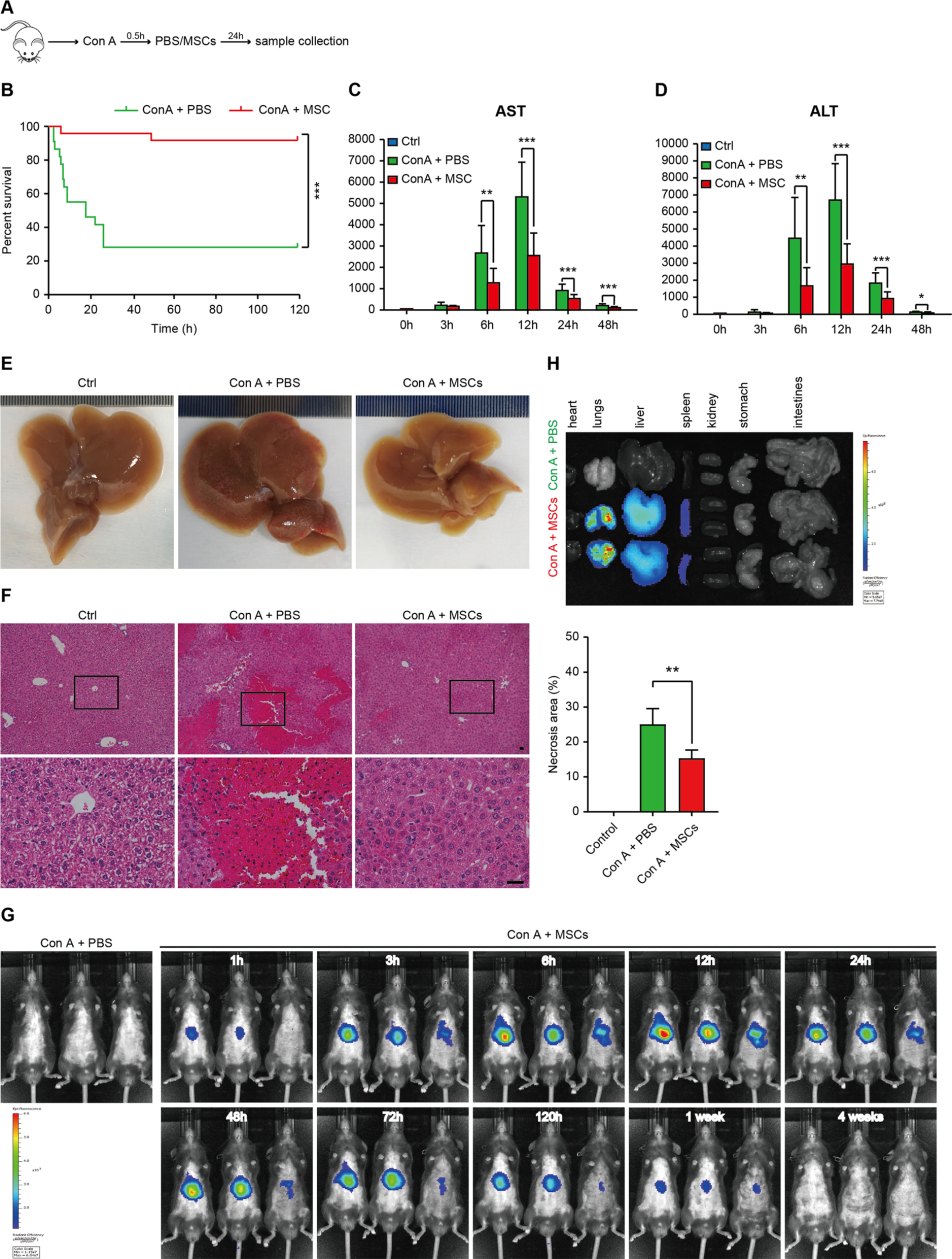
hWJ-MSCs effectively protected mouse from Con A-induced fulminant hepatitis. **A** Schematic illustration of the experimental design*.* **B** The survival curves of mice in indicated groups within 120 h after Con A administration. **C** and **D** Serum AST (**C**) and ALT (**D**) levels were measured at indicated time point after Con A treatment in indicated groups. **E** Representative gross appearance of livers 24 h after Con A treatment in indicated groups. **F** Representative H&E staining photographs (left) and quantification of necrosis areas (right, n = 5/group) of liver tissues 24 h after Con A administration in indicated groups. Scale bar = 100 μm. Magnified images of the boxed areas were shown at the bottom. **G** Live imaging of DiR-labeled MSCs at indicated time point after Con A administration using Xenogen IVIS Lumina II. PBS was used as a negative control. **H** Imaging of different organs dissected at 24 h after Con A administration in indicated groups using Xenogen IVIS Lumina II. Data in (**B**–**D**) are shown as mean ± SD (*n* = 9–12/group) with the indicated significance (*p < 0.05, **p < 0.01, ***p < 0.001)

hWJ-MSCs demonstrated significant protection on Con A-induced death (Fig. 1A, B). A sub-lethal dose of Con A (15 mg/kg) was then administrated to study the mechanism of hWJ-MSCs-mediated protection on Con A-induced liver injury. The hWJ-MSCs group showed significant decrease of serum ALT and AST activities (two liver injury markers) at 6, 12, 24, and 48 h after Con A administration, indicating an immediate, effective improvement of Con A-induced liver injury by hWJ-MSCs (Fig. 1C, D). Gross liver appearance and histological analysis at 24 h showed that hWJ-MSCs effectively ameliorated Con A-induced hepatocyte necrosis, disseminated hemorrhage and collagen deposition (Fig. 1E, F, and Additional file 1: Figure S1D). Live imaging showed that transplanted MSCs mainly localized in the liver and the lungs (Fig. 1G, H). These results confirm that hWJ-MSCs effectively protected the mouse from Con A-induced liver injury in vivo.

### hWJ-MSCs suppressed the activation of CD3^+^ hepatic T cells in Con A-induced fulminant hepatitis

**Fig. 2:**
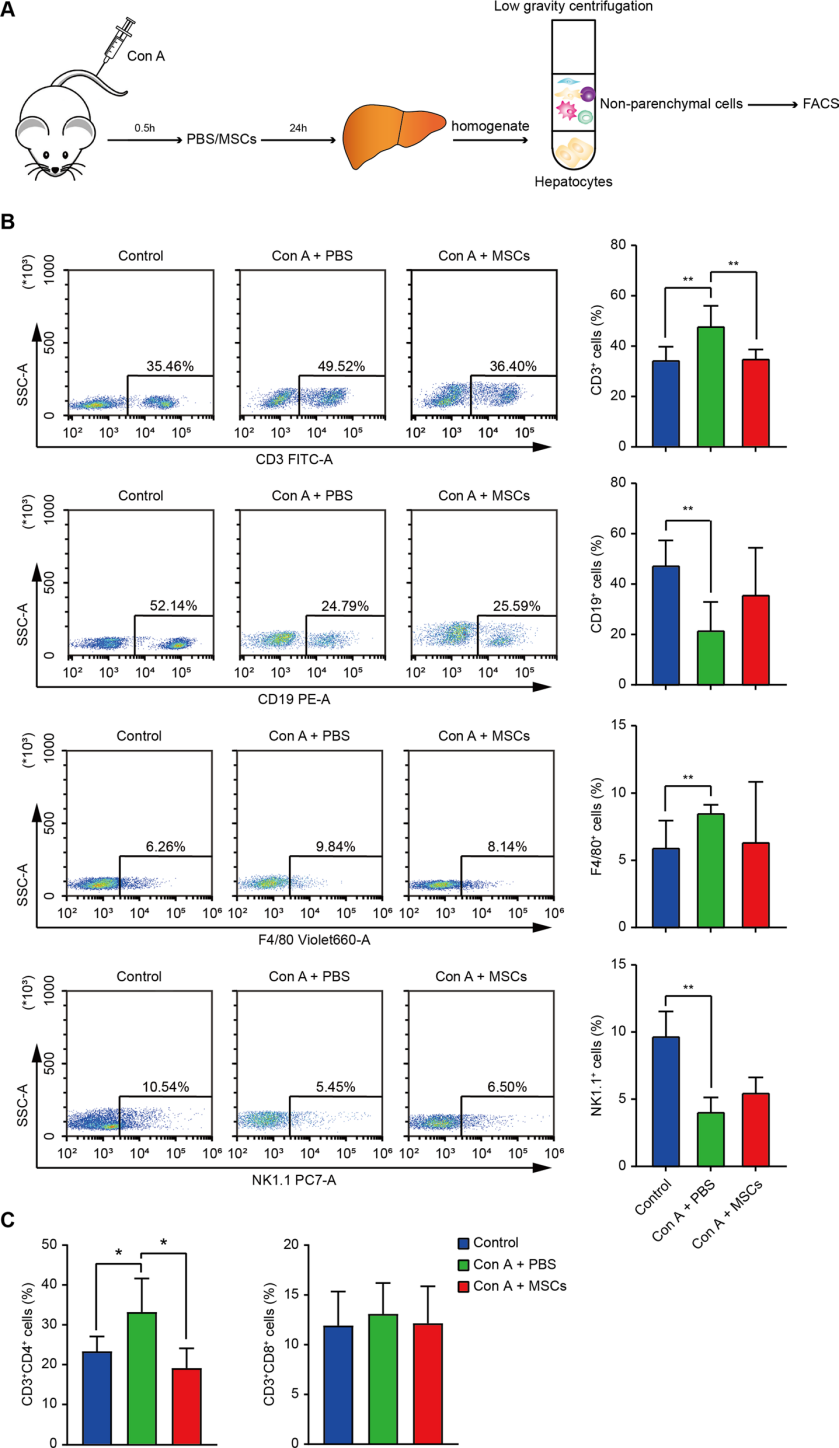
hWJ-MSCs suppressed the activation of CD3^+^ hepatic T cells in Con A-induced fulminant hepatitis. **A** Schematic illustration of the experimental design. **B** Representative plots (left) and quantification (right) of flow cytometry analysis for CD3, CD19, F4/80 and NK1.1 expression in CD45^+^ liver non-parenchymal cells 24 h after Con A administration in indicated groups. **C** Quantification of flow cytometry analysis for CD3^+^CD4^+^ and CD3^+^CD8^+^ T cell ratio in CD45^+^ liver non-parenchymal cells 24 h after Con A administration in indicated groups. Data in **B** and **C** is shown as mean ± SD (n = 5–9/group) with the indicated significance (*p < 0.05, **p < 0.01)

The Con A-induced liver injury model is characterized by lymphocyte-induced liver damage, especially CD4^+^ T lymphocytes activation and infiltration in the liver [41]. Therefore, this model is considered a suitable mouse model for studying the pathological mechanisms of liver damage due to, for example, human viral hepatitis and autoimmune liver disease [20]. However, activation of other immune cells (including B cells, macrophages, NK cells, and NKT cells) has also contributed to Con A-induced hepatitis [21, 42–44]. To determine which immune cells hWJ-MSCs modulate in the liver, liver non-parenchymal cells of control, Con A + PBS group, and Con A + hWJ-MSCs group were isolated 24 h after Con A administration. Flow cytometry analysis was undertaken to determine which subpopulations were changed by hWJ-MSCs (Fig. 2A). The results showed that the number of CD3^+^ T cells and F4/80 + macrophages was increased after Con A administration, while CD19^+^ B lymphocytes and NK1.1^+^ NK/NKT cells were decreased (Fig. 2B). However, only the CD3^+^ T cell (especially CD3^+^CD4^+^ T cell) ratio recovered after the treatment with hWJ-MSCs (Fig. 2B, C). These results infer that hWJ-MSCs alleviate Con A-induced hepatitis primarily through suppressing hepatic CD3^+^ T cells.

### Transcriptional analysis of mechanisms hWJ-MSCs alleviate Con A-induced fulminant hepatitis in vivo

**Fig. 3:**
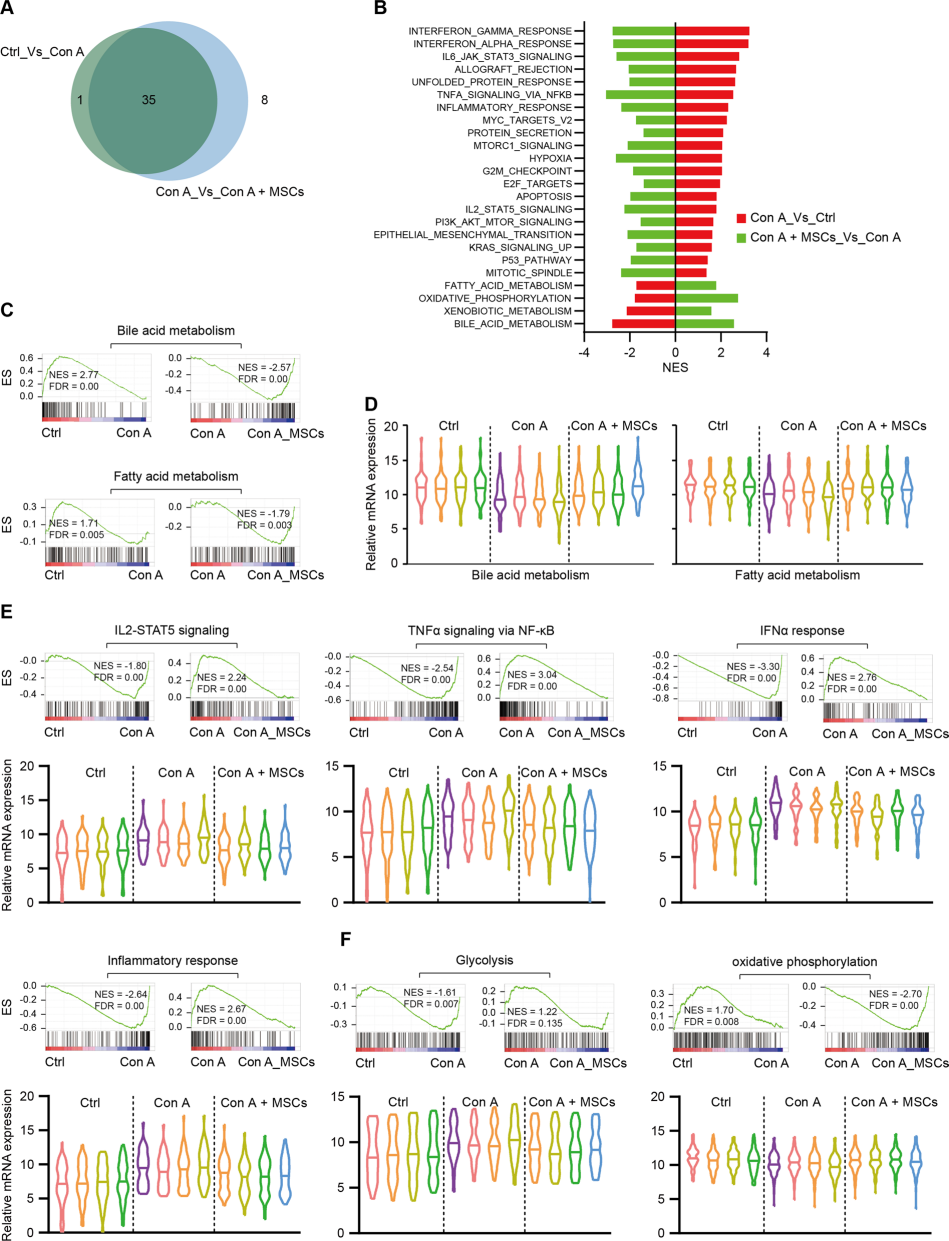
RNA sequencing of liver tissues showing alleviation of Con A-induced mouse liver injury by hWJ-MSCs. **A** Venn diagrams showing significantly enriched gene pathways of Control (Ctrl, also here after in similar experiments) versus Con A group and Con A versus Con A + MSCs group by GSEA analysis*.* **B** Bar plot showing normalized enrichment score (NES) of significantly changed GSEA pathways of Control versus Con A group and Con A versus Con A + MSCs group in GSEA analysis. **C** GSEA analysis showing bile acid metabolism (upper) and fatty acid metabolism (lower), were enriched in indicated groups. **D** Violin plots showing relative mRNA level of genes involved in bile acid metabolism (left) and fatty acid metabolism (right) in indicated groups, 4 individuals were shown for each group. **E** and **F** GSEA analysis showing enrichment of indicated gene sets (upper) and violin plots showing relative mRNA level of involved genes in indicated groups (lower). NES, normalized enrichment score; FDR, false discovery rate; GSEA, gene set enrichment analysis

The RNA sequencing for liver tissues of the control group, Con A + PBS group, and Con A + hWJ-MSCs group 24 h after Con A administration was performed, to further characterise the mechanisms via which hWJ-MSCs alleviate Con A-induced hepatitis (Additional file 3: Table S2). Gene set enrichment analysis (GSEA) showed that almost all (35/36) pathways significantly changed in the Con A + PBS group versus the control group were recovered in the Con A + hWJ-MSCs group (Fig. 3A). We found that crucial liver functions, such as bile acid metabolism, xenobiotic metabolism, and fatty acid metabolism, were repressed in the Con A + PBS group and reconstituted in the Con A + hWJ-MSCs group (Fig. 3B–D). Also, inflammatory response pathways including IL2-STAT5, TNFα signaling via NF-κB, IFNα, and IFNγ response were among the most significantly enriched gene pathways in the Con A + PBS group, whereas repressed in the Con A + hWJ-MSCs group (Fig. 3B, E). Consistent with the phenomenon of massive hepatocyte necrosis and activation of immune cells, apoptosis pathway and cell cycle-related pathways (including E2F targets, G2M checkpoints, and Myc targets) were activated in the Con A + PBS group, and both pathways returned to the resting state in the Con A + hWJ-MSCs group (Additional file 1: Figure S2A and 2B), consistent with the observation of decreased hepatocyte necrosis (Fig. 1F) and suppressed T cell infiltration in the liver (Fig. 2B) after hWJ-MSCs treatment. Notably, we also observed significant changes in other metabolism pathways, including activation of glycolysis, mTORC1, and hypoxia response after Con A administration and corresponding recovery following hWJ-MSCs infusion. However, the oxidative phosphorylation pathways showed the opposite tendency (Fig. 3F, Additional file 1: Figure S2C). In total, 562/674 upregulated and 546/726 downregulated genes in Con A + hWJ-MSCs group compared to Con A + PBS group showed opposite expression tendencies in the Con A + PBS group versus the control group (Additional file 1: Figure S2D and 2E), demonstrating significant molecular changes following hWJ-MSC treatment. GO and KEGG pathway analysis of differentially expressed genes (DEGs) showed similar results to the GSEA analysis, confirming metabolism and inflammatory pathways are the primary pathways mediating the therapeutic effects of hWJ-MSCs on Con A-induced hepatitis (Additional file 1: Figure S3 and S4).

### Bioinformatic analysis of mechanisms hWJ-MSCs inhibit the activation and proliferation of T cells in vitro

**Fig. 4:**
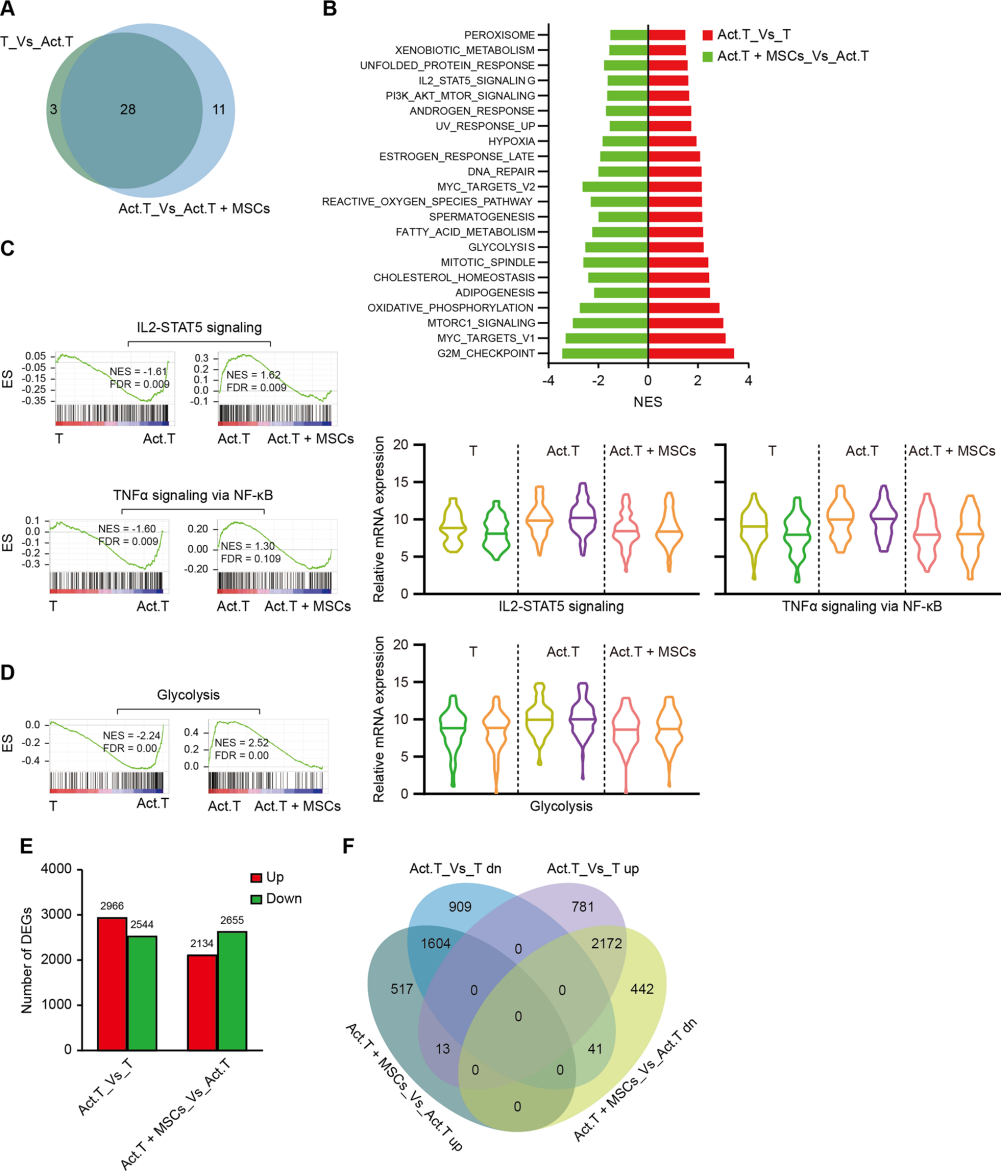
RNA sequencing of human CD3^+^ T cells showing inhibition of T cell immunity by hWJ-MSCs in vitro. **A** Venn diagrams showing significantly enriched gene pathways of naïve T cells (T) versus activated T cells (Act.T) group and Act.T versus Act.T + MSCs group by GSEA analysis. **B** Bar plot showing normalized enrichment score (NES) of significantly changed GSEA pathways of T versus Act.T group and Act.T versus Act.T + MSCs group in GSEA analysis. **C** GSEA analysis showing IL2-STAT5 and TNFα signaling via NF-κB pathways were enriched in indicated groups (left). Violin plots showing relative mRNA level of genes involved in IL2-STAT5 and TNFα signaling via NF-κB in indicated groups, 2 replicates were shown for each group (right). **D** GSEA analysis showing glycolysis enrichment in indicated groups (left) and violin plots showing relative mRNA level of genes involved in glycolysis in indicated groups (right). **E** Summary of number of differentially expressed genes (DEGs) of Act.T versus T group and Act.T + MSCs versus Act.T group. **F** Venn diagrams of the DEGs among different groups

Since Con A-induced fulminant hepatitis is mainly T cell-mediated hepatic injury, and treatment with hWJ-MSCs could ameliorate Con A-induced liver injury principally by reducing CD3^+^ T cell infiltration (Fig. 2B), to further investigate the mechanisms by which hWJ-MSCs function, we performed RNA sequencing analysis of naïve T cells, activated T cells, and activated T cells co-cultured with hWJ-MSCs (Additional file 4: Table S3). Like liver tissues, most (28/31) of the significantly enriched pathways comparing T cells versus activated T cells were recovered when comparing activated T cells versus activated T cells co-cultured with hWJ-MSCs (Fig. 4A), indicating a strong suppression of T cell immunity by hWJ-MSCs. GSEA analysis showed that similar to Con A-induced liver injury models, inflammatory pathways, such as IL-2-STAT5, TNFα signaling via NF-κB (Fig. 4B, C), cell cycle-related pathways (Additional file 1: Figure S5A), and metabolism pathways (including glucose metabolic process, hypoxia, and mTORC1 signaling) (Fig. 4D, Additional file 1: Figure S5B) were enriched in activated T cells and repressed in activated T cells co-cultured with hWJ-MSCs. Interestingly, unlike in liver tissues, oxidative phosphorylation pathways were activated in activate T cells (Additional file 1: Figure S5C), consistent with previous reports that both aerobic glycolysis and oxidative phosphorylation were increased in activated T cells [45]. Most of the differentially regulated genes after T cell activation recovered in activated T cells co-cultured with hWJ-MSCs (Fig. 4E, F). The KEGG pathway analysis of these differentially regulated genes confirmed significant metabolic changes among all three groups (Additional file 1: Figure S5D).

### NF-κB signaling and glycolysis are shared pathways between the in vivo and in vitro models

**Fig. 5:**
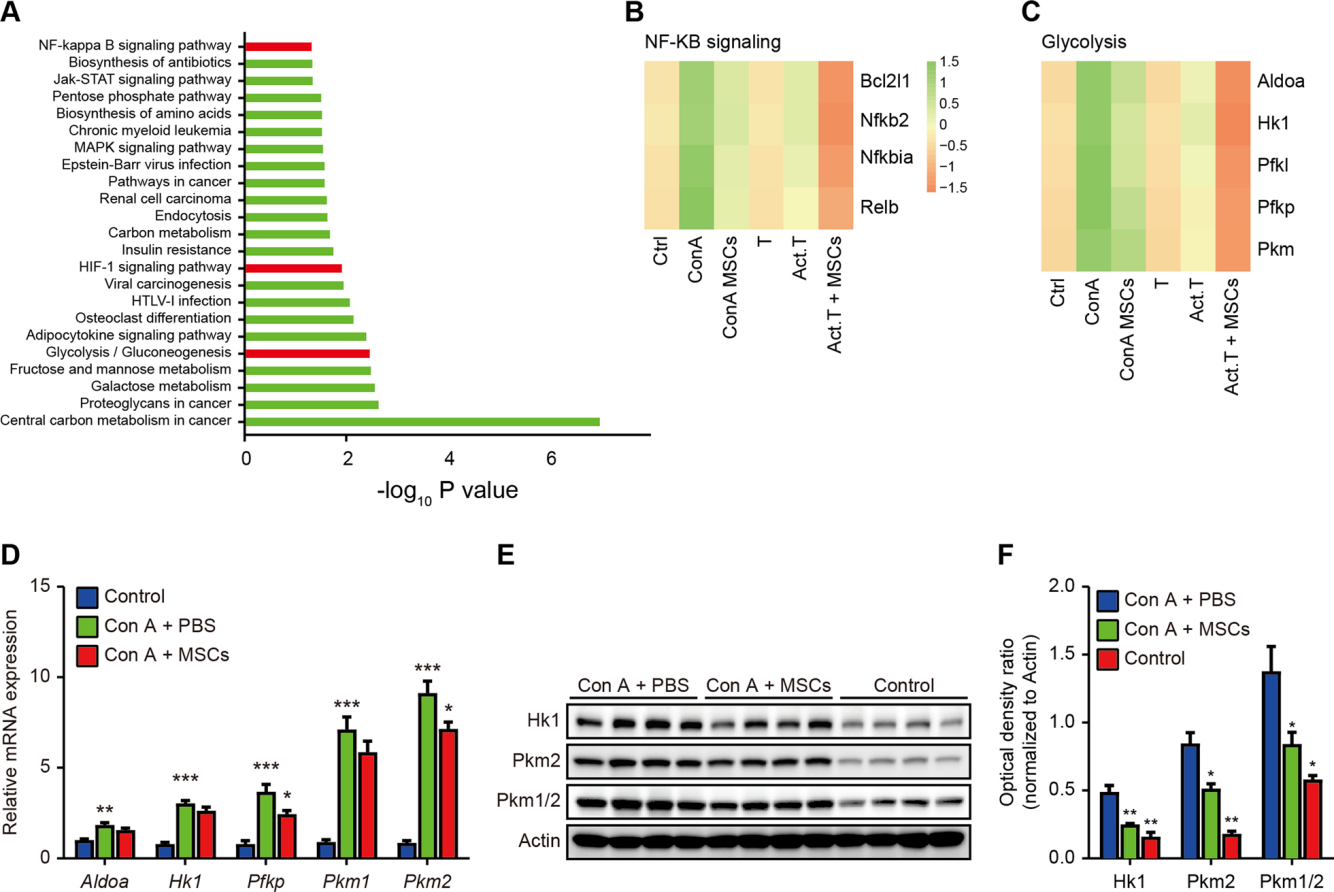
NF-κB signaling and glycolysis are shared pathways between in vivo and in vitro models. **A** KEGG pathway analysis of the overlapped DEGs between Con A + hWJ-MSCs versus Con A + PBS group, Con A + PBS versus control group, activated T cells co-cultured with hWJ-MSCs versus activated T cells and activated T cells versus naïve T cells. **B** and **C** Heatmap showing expression of key genes of NF-κB signaling pathway (**B**) and glycolysis (**C**) in indicated groups. **D** RT-qPCR for key genes involved in glycolysis in mouse liver tissues in indicated groups (*n* = 5/group). **E** and **F** Western blotting (**E**) and corresponding densitometry analysis **(F)** for key genes involved in glycolysis in mouse liver tissues in indicated groups (*n* = 4/group). Data in **D** and **F** is shown as mean ± SD with the indicated significance (*p < 0.05, **p < 0.01, ***p < 0.001). KEGG, Kyoto Encyclopaedia of Genes and Genomes

The overlapped genes of the four categories were considered and analyzed: (1) downregulated in Con A + hWJ-MSCs versus Con A + PBS group, (2) upregulated in Con A + PBS versus control group, (3) downregulated in activated T cells co-cultured with hWJ-MSCs versus activated T cells, and (4) upregulated in activated T cells versus naïve T cells. 89 genes met the criteria, and the KEGG pathway analysis showed that the NF-κB signaling pathway, HIF-1α signaling, and glycolysis pathways were among the most significantly enriched pathways (Fig. 5A). Key genes involved in NF-κB signaling and glycolysis were upregulated in Con A-treated mouse liver tissues and activated human T cells, while all were repressed following treatment with hWJ-MSCs. As HIF-1α signaling could directly induce glycolysis under hypoxic conditions [46, 47], these results confirm that NF-κB signaling and glycolysis metabolic processes are two important pathways mediating the therapeutic function of hWJ-MSCs both in Con A-induced fulminant hepatitis in vivo and T cell activation in vitro (Fig. 5B, C). RT-qPCR and western blotting analysis of liver tissues confirmed that Con A administration indeed induced key enzymes involved in glycolysis, especially PKM2, the enzyme responsible for the last step of glycolysis, and MSCs treatment partially blunted this effect (Fig. 5D–F).

## Discussion

Ease of isolation and gene modification, low immunogenicity, chemotaxis to damaged tissues, potent immunoregulatory functions, and many other advantages infer that MSCs have tremendous potential in cell therapy and regenerative medicine [7, 48–51]. In the present study, human umbilical cord Wharton's jelly-derived MSCs were used to treat fulminant hepatitis. The results confirmed that hWJ-MSCs have significant therapeutic effects on Con A-induced fulminant hepatitis in vivo and potently inhibit T cell immunity in vitro and are as functional as MSCs from other origins [29, 31, 32].

Concanavalin A (Con A) is a plant lectin extracted from Brazilian rubber Canavalia. Con A-induced liver injury is a commonly used murine liver injury model considered suitable for studying the pathological mechanisms of human viral hepatitis and autoimmune liver disease. The changes in hemodynamics, morphology, and biochemical metabolism are similar to human viral hepatitis and autoimmune liver disease. In Con A-induced liver injury, lymphocyte (especially CD4^+^ T cell) activation and infiltration play a central role in inducing liver damage [20, 21, 42, 44, 52–54]. Con A can activate macrophages in the liver sinusoid to produce cytokines, directly damaging liver cells [55], or activate T lymphocytes to produce cytokines and infiltrate the liver, leading to immune liver damage [56]. Previous studies reported that MSCs from different origins could alleviate Con A-induced hepatitis by acting on different types of immune cells, including switching Con A-stimulated macrophages to the M2 phenotype by bone marrow-derived MSCs [29], suppression of myeloid-lineage cells and CD4^+^ T cell by adipose-derived MSCs [32], suppression of T cells by both bone marrow-derived MSCs and tonsil-derived MSCs [28, 31], and suppression of NKT cells by bone marrow-derived MSCs [26, 30]. Interestingly, our results demonstrated that hWJ-MSCs alleviate Con A-induced fulminant hepatitis primarily by suppressing hepatic CD3^+^ T cells with no significant change of macrophages, CD19^+^ B lymphocytes and NK1.1^+^ NK/NKT cells. These results indicated that MSCs from different origins might exert therapeutic outcomes through disparate mechanisms.

To further elucidate the regulatory mechanisms by which hWJ-MSCs protect Con A-induced fulminant hepatitis, RNA sequencing analysis of liver tissues was undertaken. As expected, noticeable improvement in liver functions was observed following hWJ-MSCs treatment, such as fatty acid metabolism, bile acid metabolism, and xenobiotic metabolism. Furthermore, inflammatory response and cell cycle-related pathways were significantly inhibited by hWJ-MSCs administration. Interestingly, we also observed the induction of glycolysis by Con A and its suppression after hWJ-MSCs administration. Notably, the changes of inflammatory response pathway, cell cycle-related pathway, and glycolysis were recapitulated in T cells activated in vitro and co-cultured with hWJ-MSCs.

Analysis of merged DEGs in mouse liver tissues and human T cells showed that NF-κB signaling and glycolysis were two critical pathways mediating the therapeutic function of hWJ-MSCs both in Con A-induced fulminant hepatitis in vivo and T cell activation in vitro. NF-κB is a common inflammatory signaling pathway. Previous studies reported that NF-κB is activated in Con A-induced fulminant hepatitis [57, 58] and suppressed by MSCs in different cell contexts [59–62]. Glycolysis plays an essential role in T cell immunity [63–67]. However, to the best of our knowledge, no previous studies have detailed the involvement of glycolysis in Con A-induced liver injury. Additionally, few studies reported MSCs modulating glycolysis in target cells/tissues [68]. Our data showed that glycolysis is upregulated in Con A-treated mouse liver tissues and activated human T cells. Simultaneously, it is repressed following treatment with hWJ-MSCs in both systems, indicating that glycolysis plays an important role in mediating the therapeutic outcomes of hWJ-MSCs. Further investigations are warranted to see whether targeting glycolysis with other therapeutics will alleviate Con A-induced fulminant hepatitis. Equally of interest are future studies to determine whether and how hWJ-MSCs could mitigate other T cell-mediated tissue injuries by inhibiting glycolysis.

## Conclusion

In this study, we confirmed the potent therapeutic role of MSCs-derived from human umbilical cord Wharton's jelly on Con A-induced fulminant hepatitis, and uncovered new mechanisms that glycolysis metabolic shift mediates suppression of T cell immunity by hWJ-MSCs.

## Supplementary Information

**Additional file 1**. Supplementary Figures.

**Additional file 2**. **Table S1.** Antibodies.

**Additional file 3**. **Table S2.** Expression matrix of liver tissues analyzed by RNA-sequencing.

**Additional file 4**. **Table S3.** Expression matrix of human T cells analyzed by RNA-sequencing.

## Data Availability

All data will be made available upon reasonable request to the corresponding authors.

## Abbreviations

aGVHD: Acute graft-versus-host disease
ALT: Alanine aminotransferase
AST: Aspartate transaminase
Con A: Concanavalin A
DAVID: Database for Annotation Visualization and Integrated Discovery
DEGs: Differentially expressed genes
FBS: Fetal bovine serum
GEO: Gene expression omnibus
GO: Gene ontology
GSEA: Gene set enrichment analysis
H&E: Hematoxylin and eosin
hWJ-MSCs: Human umbilical cord Wharton's jelly-derived MSCs
IBMX: Isobutylmethylxanthine
KEGG: Kyoto encyclopaedia of genes and genomes
MSCs: Mesenchymal stem cells
PBMC: Peripheral blood mononuclear cells
PHA: Phytohaemagglutinin
PKM1/2: Pyruvate kinase M1/2
PKM2: Pyruvate kinase M2
PSR: Picrosirius red

## References

[1] Ichai P, Samuel D (2008). Etiology and prognosis of fulminant hepatitis in adults. Liver Transpl.

[2] Kern S, Eichler H, Stoeve J, Kluter H, Bieback K (2006). Comparative analysis of mesenchymal stem cells from bone marrow, umbilical cord blood, or adipose tissue. Stem Cells.

[3] Campagnoli C, Roberts IA, Kumar S, Bennett PR, Bellantuono I, Fisk NM (2001). Identification of mesenchymal stem/progenitor cells in human first-trimester fetal blood, liver, and bone marrow. Blood.

[4] Chen L, Qu J, Mei Q, Chen X, Fang Y, Chen L (2021). Small extracellular vesicles from menstrual blood-derived mesenchymal stem cells (MenSCs) as a novel therapeutic impetus in regenerative medicine. Stem Cell Res Ther.

[5] Ling L, Feng X, Wei T, Wang Y, Wang Y, Wang Z (2019). Human amnion-derived mesenchymal stem cell (hAD-MSC) transplantation improves ovarian function in rats with premature ovarian insufficiency (POI) at least partly through a paracrine mechanism. Stem Cell Res Ther.

[6] Antony AK, Rodby K, Tobin MK, O'Connor MI, Pearl RK, DiPietro LA (2013). Composite tissue allotransplantation and dysregulation in tissue repair and regeneration: a role for mesenchymal stem cells. Front Immunol.

[7] De Becker A, Riet IV (2016). Homing and migration of mesenchymal stromal cells: how to improve the efficacy of cell therapy?. World J Stem Cells.

[8] Singer NG, Caplan AI (2011). Mesenchymal stem cells: mechanisms of inflammation. Annu Rev Pathol.

[9] Peng Y, Chen X, Liu Q, Zhang X, Huang K, Liu L (2015). Mesenchymal stromal cells infusions improve refractory chronic graft versus host disease through an increase of CD5+ regulatory B cells producing interleukin 10. Leukemia.

[10] Ghoryani M, Shariati-Sarabi Z, Tavakkol-Afshari J, Mohammadi M (2020). The sufficient immunoregulatory effect of autologous bone marrow-derived mesenchymal stem cell transplantation on regulatory T cells in patients with refractory rheumatoid arthritis. J Immunol Res.

[11] Lightner AL, Dozois EJ, Dietz AB, Fletcher JG, Friton J, Butler G (2020). Matrix-delivered autologous mesenchymal stem cell therapy for refractory rectovaginal Crohn's fistulas. Inflamm Bowel Dis.

[12] Bulati M, Miceli V, Gallo A, Amico G, Carcione C, Pampalone M (2020). The immunomodulatory properties of the human amnion-derived mesenchymal stromal/stem cells are induced by INF-gamma produced by activated lymphomonocytes and are mediated by cell-to-cell contact and soluble factors. Front Immunol.

[13] Poggi A, Zocchi MR (2019). Immunomodulatory properties of mesenchymal stromal cells: still unresolved "Yin and Yang". Curr Stem Cell Res Ther.

[14] Shi Y, Wang Y, Li Q, Liu K, Hou J, Shao C (2018). Immunoregulatory mechanisms of mesenchymal stem and stromal cells in inflammatory diseases. Nat Rev Nephrol.

[15] Ren G, Zhang L, Zhao X, Xu G, Zhang Y, Roberts AI (2008). Mesenchymal stem cell-mediated immunosuppression occurs via concerted action of chemokines and nitric oxide. Cell Stem Cell.

[16] Soleymaninejadian E, Pramanik K, Samadian E (2012). Immunomodulatory properties of mesenchymal stem cells: cytokines and factors. Am J Reprod Immunol.

[17] Lin BL, Chen JF, Qiu WH, Wang KW, Xie DY, Chen XY (2017). Allogeneic bone marrow-derived mesenchymal stromal cells for hepatitis B virus-related acute-on-chronic liver failure: a randomized controlled trial. Hepatology.

[18] Shi M, Zhang Z, Xu R, Lin H, Fu J, Zou Z (2012). Human mesenchymal stem cell transfusion is safe and improves liver function in acute-on-chronic liver failure patients. Stem Cells Transl Med.

[19] Sun K, Xie X, Xie J, Jiao S, Chen X, Zhao X (2014). Cell-based therapy for acute and chronic liver failures: distinct diseases, different choices. Sci Rep.

[20] Tiegs G, Hentschel J, Wendel A (1992). A T cell-dependent experimental liver injury in mice inducible by concanavalin A. J Clin Investig.

[21] Kusters S, Gantner F, Kunstle G, Tiegs G (1996). Interferon gamma plays a critical role in T cell-dependent liver injury in mice initiated by concanavalin A. Gastroenterology.

[22] Heymann F, Hamesch K, Weiskirchen R, Tacke F (2015). The concanavalin A model of acute hepatitis in mice. Lab Anim.

[23] Ye T, Wang T, Yang X, Fan X, Wen M, Shen Y (2018). Comparison of concanavalin A-induced murine autoimmune hepatitis models. Cell Physiol Biochem.

[24] Zhou L, Liu S, Wang Z, Yao J, Cao W, Chen S (2019). Bone marrow-derived mesenchymal stem cells modified with Akt1 ameliorates acute liver GVHD. Biol Proced Online.

[25] Tamura R, Uemoto S, Tabata Y (2016). Immunosuppressive effect of mesenchymal stem cell-derived exosomes on a concanavalin A-induced liver injury model. Inflamm Regen.

[26] Zhu X, He B, Zhou X, Ren J (2013). Effects of transplanted bone-marrow-derived mesenchymal stem cells in animal models of acute hepatitis. Cell Tissue Res.

[27] Gu Y, Ding X, Huang J, Xue M, Zhang J, Wang Q (2018). The deubiquitinating enzyme UCHL1 negatively regulates the immunosuppressive capacity and survival of multipotent mesenchymal stromal cells. Cell Death Dis.

[28] Han X, Yang Q, Lin L, Xu C, Zheng C, Chen X (2014). Interleukin-17 enhances immunosuppression by mesenchymal stem cells. Cell Death Differ.

[29] Lee KC, Lin HC, Huang YH, Hung SC (2015). Allo-transplantation of mesenchymal stem cells attenuates hepatic injury through IL1Ra dependent macrophage switch in a mouse model of liver disease. J Hepatol.

[30] Gazdic M, Simovic Markovic B, Vucicevic L, Nikolic T, Djonov V, Arsenijevic N (2018). Mesenchymal stem cells protect from acute liver injury by attenuating hepatotoxicity of liver natural killer T cells in an inducible nitric oxide synthase- and indoleamine 2,3-dioxygenase-dependent manner. J Tissue Eng Regen Med.

[31] Ryu KH, Kim SY, Kim YR, Woo SY, Sung SH, Kim HS (2014). Tonsil-derived mesenchymal stem cells alleviate concanavalin A-induced acute liver injury. Exp Cell Res.

[32] Higashimoto M, Sakai Y, Takamura M, Usui S, Nasti A, Yoshida K (2013). Adipose tissue derived stromal stem cell therapy in murine ConA-derived hepatitis is dependent on myeloid-lineage and CD4+ T-cell suppression. Eur J Immunol.

[33] Kubo N, Narumi S, Kijima H, Mizukami H, Yagihashi S, Hakamada K (2012). Efficacy of adipose tissue-derived mesenchymal stem cells for fulminant hepatitis in mice induced by concanavalin A. J Gastroenterol Hepatol.

[34] Nasti A, Sakai Y, Seki A, Buffa GB, Komura T, Mochida H (2017). The CD45(+) fraction in murine adipose tissue derived stromal cells harbors immune-inhibitory inflammatory cells. Eur J Immunol.

[35] Wang W, Guo H, Li H, Yan Y, Wu C, Wang X (2018). Interleukin-35 gene-modified mesenchymal stem cells protect concanavalin A-induced fulminant hepatitis by decreasing the interferon gamma level. Hum Gene Ther.

[36] Weiss ML, Anderson C, Medicetty S, Seshareddy KB, Weiss RJ, VanderWerff I (2008). Immune properties of human umbilical cord Wharton's jelly-derived cells. Stem Cells.

[37] Weiss ML, Medicetty S, Bledsoe AR, Rachakatla RS, Choi M, Merchav S (2006). Human umbilical cord matrix stem cells: preliminary characterization and effect of transplantation in a rodent model of Parkinson's disease. Stem Cells.

[38] Zhuang Q, Li W, Benda C, Huang Z, Ahmed T, Liu P (2018). NCoR/SMRT co-repressors cooperate with c-MYC to create an epigenetic barrier to somatic cell reprogramming. Nat Cell Biol.

[39] Subramanian A, Tamayo P, Mootha VK, Mukherjee S, Ebert BL, Gillette MA (2005). Gene set enrichment analysis: a knowledge-based approach for interpreting genome-wide expression profiles. Proc Natl Acad Sci U S A.

[40] Love MI, Huber W, Anders S (2014). Moderated estimation of fold change and dispersion for RNA-seq data with DESeq2. Genome Biol.

[41] Hong F, Jaruga B, Kim WH, Radaeva S, El-Assal ON, Tian Z (2002). Opposing roles of STAT1 and STAT3 in T cell-mediated hepatitis: regulation by SOCS. J Clin Investig.

[42] Erhardt A, Biburger M, Papadopoulos T, Tiegs G (2007). IL-10, regulatory T cells, and Kupffer cells mediate tolerance in concanavalin A-induced liver injury in mice. Hepatology.

[43] Schumann J, Wolf D, Pahl A, Brune K, Papadopoulos T, van Rooijen N (2000). Importance of Kupffer cells for T-cell-dependent liver injury in mice. Am J Pathol.

[44] Takeda K, Hayakawa Y, Van Kaer L, Matsuda H, Yagita H, Okumura K (2000). Critical contribution of liver natural killer T cells to a murine model of hepatitis. Proc Natl Acad Sci U S A.

[45] Chang CH, Curtis JD, Maggi LB, Faubert B, Villarino AV, O'Sullivan D (2013). Posttranscriptional control of T cell effector function by aerobic glycolysis. Cell.

[46] Codo AC, Davanzo GG, Monteiro LB, de Souza GF, Muraro SP, Virgilio-da-Silva JV (2020). Elevated glucose levels favor SARS-CoV-2 infection and monocyte response through a HIF-1alpha/glycolysis-dependent axis. Cell Metab.

[47] Cheng SC, Quintin J, Cramer RA, Shepardson KM, Saeed S, Kumar V (2014). mTOR- and HIF-1alpha-mediated aerobic glycolysis as metabolic basis for trained immunity. Science.

[48] Pittenger MF, Mackay AM, Beck SC, Jaiswal RK, Douglas R, Mosca JD (1999). Multilineage potential of adult human mesenchymal stem cells. Science.

[49] Le Blanc K, Mougiakakos D (2012). Multipotent mesenchymal stromal cells and the innate immune system. Nat Rev Immunol.

[50] Uccelli A, Moretta L, Pistoia V (2008). Mesenchymal stem cells in health and disease. Nat Rev Immunol.

[51] Galipeau J, Sensebe L (2018). Mesenchymal stromal cells: clinical challenges and therapeutic opportunities. Cell Stem Cell.

[52] Kaneko Y, Harada M, Kawano T, Yamashita M, Shibata Y, Gejyo F (2000). Augmentation of Valpha14 NKT cell-mediated cytotoxicity by interleukin 4 in an autocrine mechanism resulting in the development of concanavalin A-induced hepatitis. J Exp Med.

[53] Lohse AW, Dienes HP, Meyer zum Buschenfelde KH (1998). Suppression of murine experimental autoimmune hepatitis by T-cell vaccination or immunosuppression. Hepatology.

[54] Wang HX, Liu M, Weng SY, Li JJ, Xie C, He HL (2012). Immune mechanisms of concanavalin A model of autoimmune hepatitis. World J Gastroenterol.

[55] Knolle PA, Gerken G, Loser E, Dienes HP, Gantner F, Tiegs G (1996). Role of sinusoidal endothelial cells of the liver in concanavalin A-induced hepatic injury in mice. Hepatology.

[56] Louis H, Le Moine O, Peny MO, Quertinmont E, Fokan D, Goldman M (1997). Production and role of interleukin-10 in concanavalin A-induced hepatitis in mice. Hepatology.

[57] Filliol A, Piquet-Pellorce C, Le Seyec J, Farooq M, Genet V, Lucas-Clerc C (2016). RIPK1 protects from TNF-alpha-mediated liver damage during hepatitis. Cell Death Dis.

[58] Zhang P, Yin Y, Wang T, Li W, Li C, Zeng X (2020). Maresin 1 mitigates concanavalin A-induced acute liver injury in mice by inhibiting ROS-mediated activation of NF-kappaB signaling. Free Radic Biol Med.

[59] Choi H, Lee RH, Bazhanov N, Oh JY, Prockop DJ (2011). Anti-inflammatory protein TSG-6 secreted by activated MSCs attenuates zymosan-induced mouse peritonitis by decreasing TLR2/NF-kappaB signaling in resident macrophages. Blood.

[60] Yagi H, Soto-Gutierrez A, Navarro-Alvarez N, Nahmias Y, Goldwasser Y, Kitagawa Y (2010). Reactive bone marrow stromal cells attenuate systemic inflammation via sTNFR1. Mol Ther.

[61] Prockop DJ, Oh JY (2012). Mesenchymal stem/stromal cells (MSCs): role as guardians of inflammation. Mol Ther.

[62] Fan B, Li C, Szalad A, Wang L, Pan W, Zhang R (2020). Mesenchymal stromal cell-derived exosomes ameliorate peripheral neuropathy in a mouse model of diabetes. Diabetologia.

[63] Lawless SJ, Kedia-Mehta N, Walls JF, McGarrigle R, Convery O, Sinclair LV (2017). Glucose represses dendritic cell-induced T cell responses. Nat Commun.

[64] Kunkl M, Sambucci M, Ruggieri S, Amormino C, Tortorella C, Gasperini C (2019). CD28 autonomous signaling up-regulates C-Myc expression and promotes glycolysis enabling inflammatory T cell responses in multiple sclerosis. Cells.

[65] Li Q, Yan Y, Liu J, Huang X, Zhang X, Kirschning C (2019). Toll-like receptor 7 activation enhances CD8+ T cell effector functions by promoting cellular glycolysis. Front Immunol.

[66] Ma EH, Verway MJ, Johnson RM, Roy DG, Steadman M, Hayes S (2019). Metabolic profiling using stable isotope tracing reveals distinct patterns of glucose utilization by physiologically activated CD8(+) T cells. Immunity.

[67] Yi HS, Kim SY, Kim JT, Lee YS, Moon JS, Kim M (2019). T-cell senescence contributes to abnormal glucose homeostasis in humans and mice. Cell Death Dis.

[68] Bottcher M, Hofmann AD, Bruns H, Haibach M, Loschinski R, Saul D (2016). Mesenchymal stromal cells disrupt mTOR-signaling and aerobic glycolysis during T-cell activation. Stem Cells.

