# Supplementary material for: Human Wharton's jelly-derived mesenchymal stem cells alleviate concanavalin A-induced fulminant hepatitis by repressing NF-κB signaling and glycolysis
Source: Stem Cell Res Ther. 2021 Sep 9;12:496. doi: 10.1186/s13287-021-02560-x (PMC8427901; doi:10.1186/s13287-021-02560-x)
Supplement: Supplementary file 1 — Additional file 1. Supplementary Figures. [file 13287_2021_2560_MOESM1_ESM.docx]

**Supplementary Figures**

**
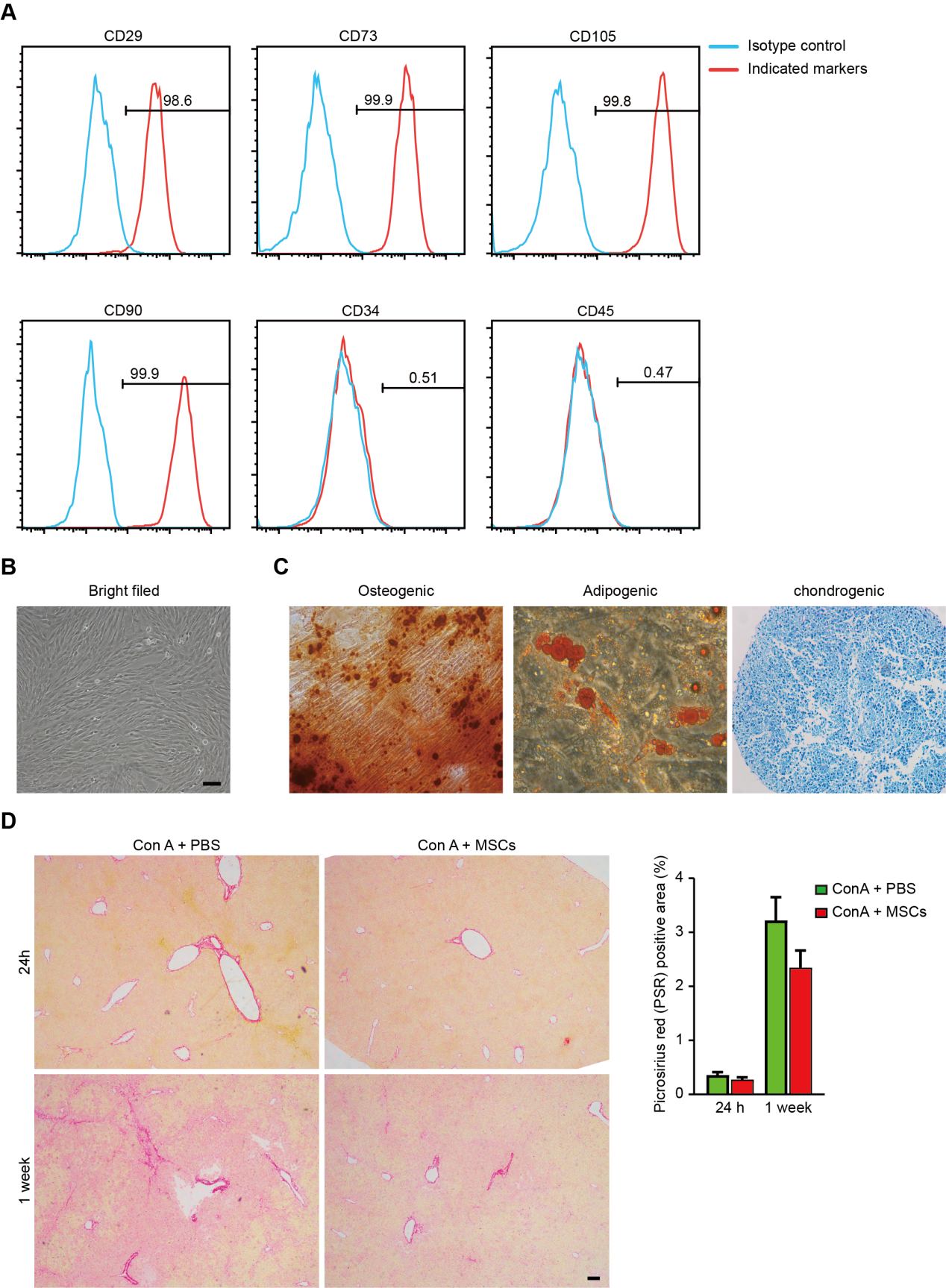
**

**Supplementary Figure 1. Characterization of hWJ-MSCs**

(A) Representative FACS plots showing hWJ-MSCs were strongly positive for CD29, CD73, CD105 and CD90, whereas negative for hematopoietic stem cell markers such as CD34 and CD45. (B) Representative morphological observation of hWJ-MSCs. Scale bar = 100 μm. (C) Representative photographs of Alizarin red, Oil red O and Toluidine blue staining for assessing the differentiation potential of hWJ-MSCs to osteoblasts, adipocytes and chondrocytes, respectively. (D) Representative Picrosirius red (PSR) staining photographs (left) and quantification of positive area (right, n = 4-5/group) of liver tissues at indicated time point after Con A administration in the indicated groups. Scale bar = 100 μm.


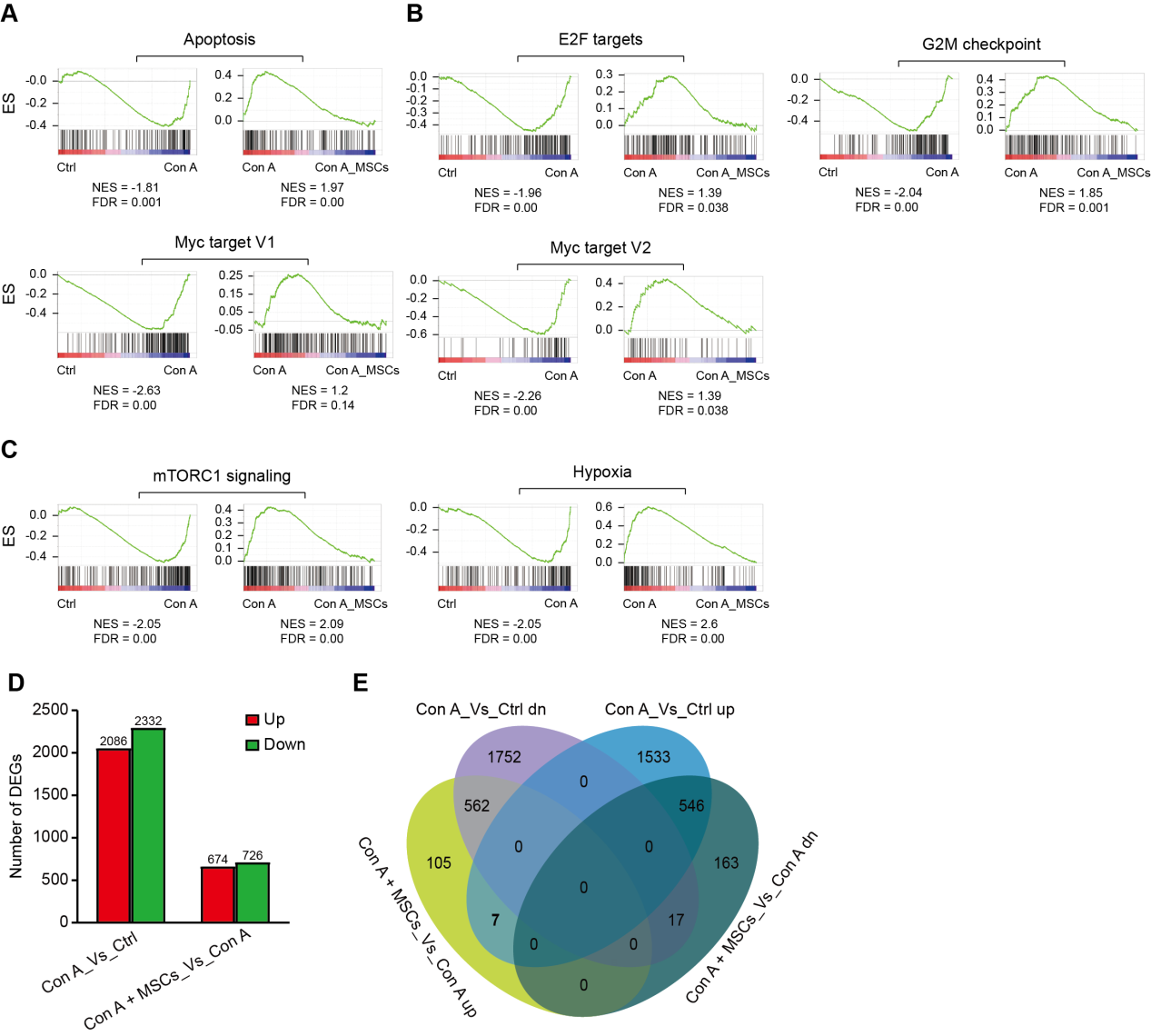


**Supplementary Figure 2. RNA-sequencing of liver tissues showing alleviation of Con A-induced mouse liver injury by hWJ-MSCs**

(A) GSEA showing that apoptosis pathway was enriched in Con A + PBS group and repressed in Con A + hWJ-MSCs group. (B) GSEA showing that cell cycle-related pathways (including E2F targets, G2M checkpoints, and Myc targets) were enriched in Con A + PBS group and repressed in Con A + hWJ-MSCs group. (C) GSEA showing that mTORC1 signaling and hypoxia pathways were enriched in Con A + PBS group and repressed in Con A + hWJ-MSCs group. (D) Number of differentially expressed genes of Con A versus Control group and Con A +MSCs versus Con A group. (E) Venn diagrams showing the number of DEGs between the Con A versus Control group and Con A +MSCs versus Con A group. NES, normalized enrichment score; FDR, false discovery rate; KEGG, Kyoto Encyclopaedia of genes and genomes.


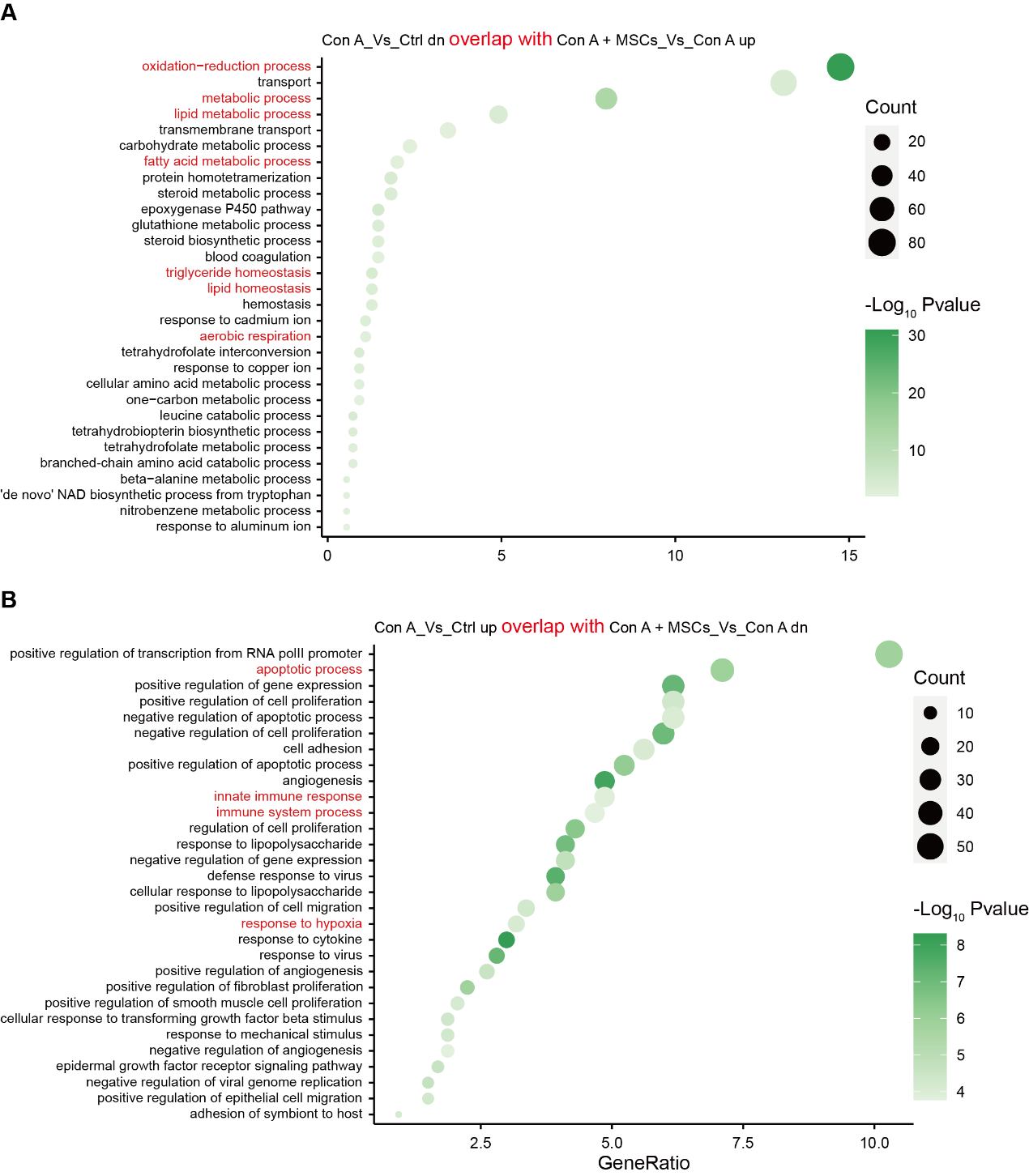


**Supplementary Figure 3. KEGG pathway analysis for mouse liver tissues of Con A-induced fulminant hepatitis with hWJ-MSCs treatment**

(A) KEGG pathway analysis of genes downregulated in Con A versus Ctrl overlapping with upregulated in Con A+MSCs versus Con A. The y-axis shows significantly enriched pathways. (B) KEGG pathway analysis of genes upregulated in Con A versus Ctrl group overlapping with downregulated in Con A + MSCs versus Con A. The y-axis shows significantly enriched pathways.


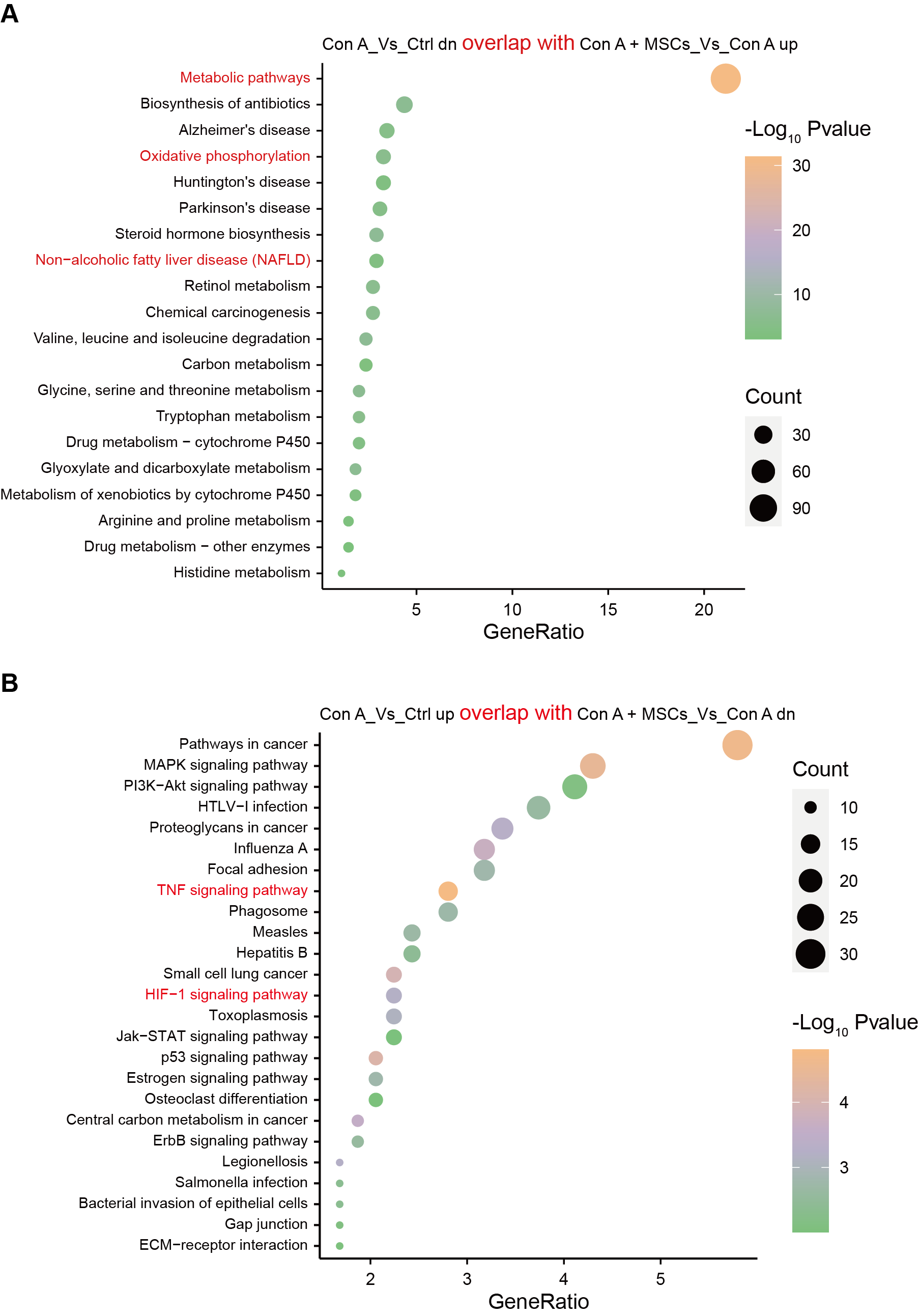


**Supplementary Figure 4. GO analysis for mouse liver tissues of Con A-induced fulminant hepatitis with hWJ-MSCs treatment**

(A) GO analysis of genes downregulated in Con A versus Ctrl overlapping with upregulated in Con A+MSCs versus Con A. The y-axis shows significantly enriched pathways. (B) GO enrichment analysis of genes upregulated in Con A versus Ctrl group overlapping with downregulated in Con A + MSCs versus Con A. The y-axis shows significantly enriched pathways.

**
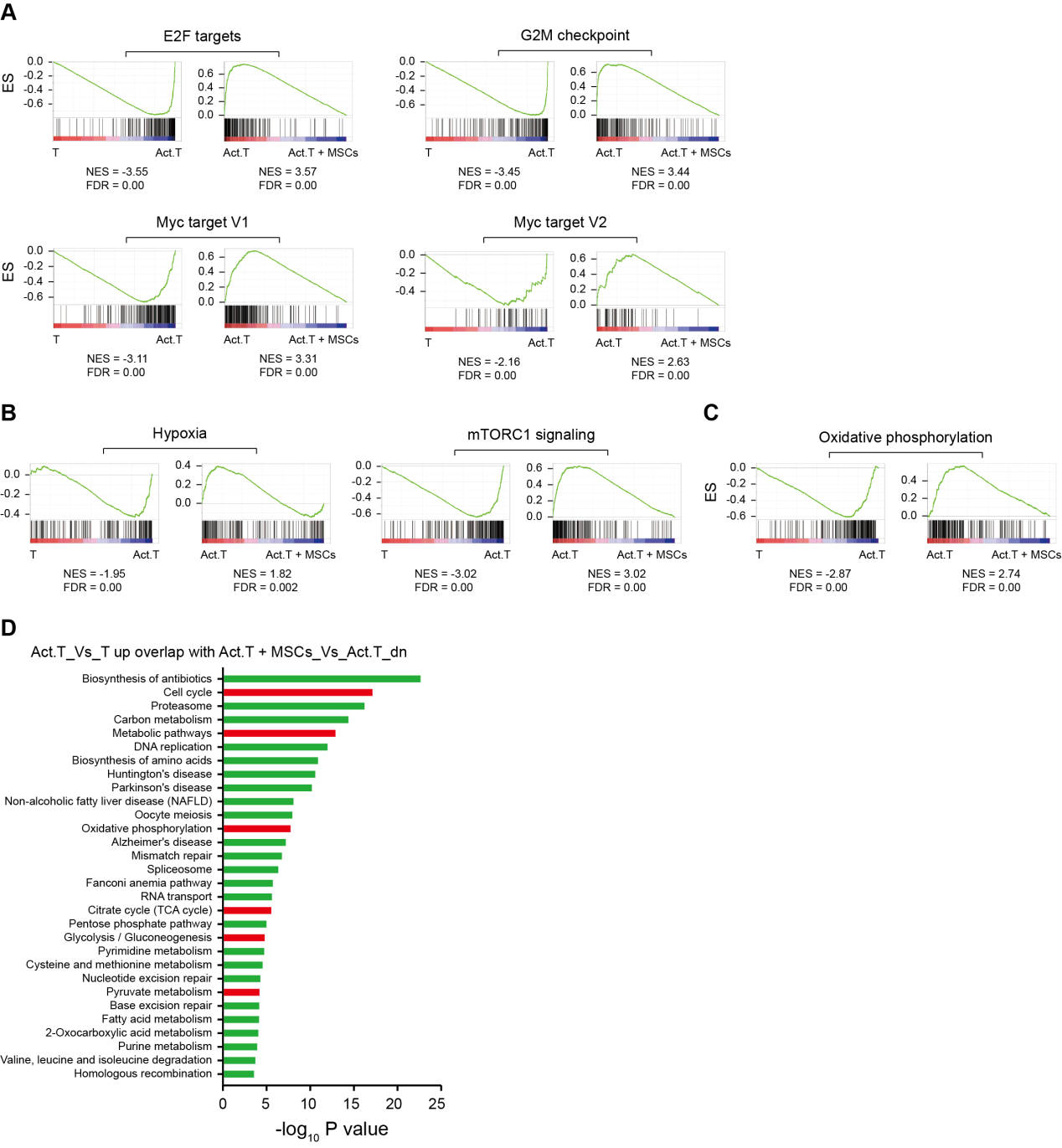
**

**Supplementary Figure 5. RNA-sequencing of human CD3^+^ T cells showing inhibition of T cell immunity by hWJ-MSCs *in vitro***

(A) GSEA showing that cell cycle-related pathways (including E2F targets, G2M checkpoints, and Myc targets) were enriched in activated T cells versus naïve T cells, whereas recovered in activated T cells co-culture with hWJ-MSCs. (B) GSEA showing the enrichment of hypoxia and mTORC1 signaling in indicated groups. (C) GSEA showing the enrichment of oxidative phosphorylation in indicated groups. (D) KEGG pathway analysis of the DEGs upregualted in Act.T versus T overlapping with downregulated in Act.T + MSCs versus Act.T group.
