# Supplementary material for: Human Wharton's jelly-derived mesenchymal stem cells alleviate concanavalin A-induced fulminant hepatitis by repressing NF-κB signaling and glycolysis
Source: Stem Cell Res Ther. 2021 Sep 9;12:496. doi: 10.1186/s13287-021-02560-x (PMC8427901; doi:10.1186/s13287-021-02560-x)
Supplement: Supplementary file 2 — Additional file 2. Table S1. Antibodies. [file 13287_2021_2560_MOESM2_ESM.docx]

**Supplementary Table 1. *Antibodies***

| **Name** | **Species reactivity** | **Application and dilution ratio** | **Supplier** | **Cat No.** | **Clone No.** |
| --- | --- | --- | --- | --- | --- |
| Anti- FOXP3 | Mouse | FACS (1:100) | BD biosciences | 563486 | R16-715 |
| Anti- CD4 | Mouse | FACS (1:100) | Biolegend | 100516 | RM4-5 |
| Anti-CD3 | Mouse | FACS (1:100) | BD biosciences | 553062 | 145-2C11 |
| Anti-CD8 | Mouse | FACS (1:100) | Biolegend | 100721 | 53-6.7 |
| Anti-CD25 | Mouse | FACS (1:100) | Biolegend | 102016 | PC61 |
| Anti-CD206 | Mouse | FACS (1:100) | Biolegend | 141704 | C068C2 |
| Anti- F4/80 | Mouse | FACS (1:100) | BD biosciences | 5655835 | T45-2342 |
| Anti-CD19 | Mouse | FACS (1:100) | Biolegend | 115508 | 6D5 |
| Anti- NK1.1 | Mouse | FACS (1:100) | Biolegend | 156513 | S17016D |
| Anti-CD29 | Human | FACS (1:100) | BD biosciences | 555443 | MAR4 |
| Anti-CD73 | Human | FACS (1:100) | BD biosciences | 561254 | AD2 |
| Anti-CD105 | Human | FACS (1:100) | BD biosciences | 561443 | 266 |
| Anti-CD90 | Human | FACS (1:100) | BD biosciences | 559869 | 5E10 |
| Anti-CD45 | Human | FACS (1:100) | BD biosciences | 557748 | HI30 |
| Anti-CD34 | Human | FACS (1:100) | BD biosciences | 560710 | 581 |
| Anti- Hexokinase I (HK1) | Mouse, Human | WB (1:1000) | Cell Signaling Technology | 2024 | C35C4 |
| Anti-Pyruvate kinase M2 (PKM2) | Mouse, Human | WB (1:1000) | Cell Signaling Technology | 4053 | D78A4 |
| Anti- Pyruvate kinase M1/2 (PKM1/2) | Mouse, Human | WB (1:1000) | Cell Signaling Technology | 3190 | C103A3 |
| Anti-β-Actin | Mouse, Human | WB (1:1000) | Cell Signaling Technology | 8457 | D6A8 |

WB, western blot.
